# Terrestrial and Aerial Laser Scanning Data Integration Using Wavelet Analysis for the Purpose of 3D Building Modeling

**DOI:** 10.3390/s140712070

**Published:** 2014-07-07

**Authors:** Michal Kedzierski, Anna Fryskowska

**Affiliations:** Department of Remote Sensing and Photogrammetry, Faculty of Civil Engineering and Geodesy, Military University of Technology, 00908 Warsaw, Poland; E-Mail: mkedzierski@wat.edu.pl

**Keywords:** terrestrial laser scanning, LiDAR, 3D model, data fusion, point cloud

## Abstract

Visualization techniques have been greatly developed in the past few years. Three-dimensional models based on satellite and aerial imagery are now being enhanced by models generated using Aerial Laser Scanning (ALS) data. The most modern of such scanning systems have the ability to acquire over 50 points per square meter and to register a multiple echo, which allows the reconstruction of the terrain together with the terrain cover. However, ALS data accuracy is less than 10 cm and the data is often incomplete: there is no information about ground level (in most scanning systems), and often around the facade or structures which have been covered by other structures. However, Terrestrial Laser Scanning (TLS) not only acquires higher accuracy data (1–5 cm) but is also capable of registering those elements which are incomplete or not visible using ALS methods (facades, complicated structures, interiors, *etc.*). Therefore, to generate a complete 3D model of a building in high Level of Details, integration of TLS and ALS data is necessary. This paper presents the wavelet-based method of processing and integrating data from ALS and TLS. Methods of choosing tie points to combine point clouds in different datum will be analyzed.

## 1. Introduction

In 2007 the number of people living in urban areas became equal to the number of those living in the countryside. The UN report State of the World Cities 2006–2007 [1] estimates that by 2030 the population of urban areas will have increased to 5 billion (about 62% of world population). In order to avoid sociological and environmental problems, local and national governments have to be prepared to appropriate “geo-reaction”, which means the assurance of appropriate tools and procedures of planning, development and the administration of urban areas using proper geoinformation systems.

### 1.1. Level of Detail

When creating 3D city models of big cities some methods of 3D building generalization are required [2]. As described in [2], the first and most popular standard, called CityGML, was developed in Germany and categorized in Levels of Details (LoDs) [3]. This standard defines LoDs for buildings models from LoDs 1 to 4. LoD 1 contains coarse building structures, LoD 2 is coarse building blocks with roof models, LoD 3 is filled with detailed building models of building facade, walls, windows, doors, *etc.*, and LoD 4 represents highly detailed architecture building models of LoD 3 but with indoor elements (stairs, installations, detailed elements, figures, sculptures, *etc.*) [2].

To generate a 3D model of the city with level of details corresponding to LoD 3 and LoD 4, specific conditions have to be fulfilled. The most important are those related to keeping details (generalization) and precision of these models.

One of the main applications of Terrestrial Laser Scanning (TLS) and Aerial Laser Scanning (ALS, sometimes also called LiDAR) is 3D city modeling. Most Digital Surface Models (DSMs) and 3D city models are generated from aerial or satellite images and LiDAR. These models are information rich about the shape of the building, but they usually do not contain many details concerning the building facade. Therefore, such models are textured with additional terrestrial or aerial images.

**Figure 1.**
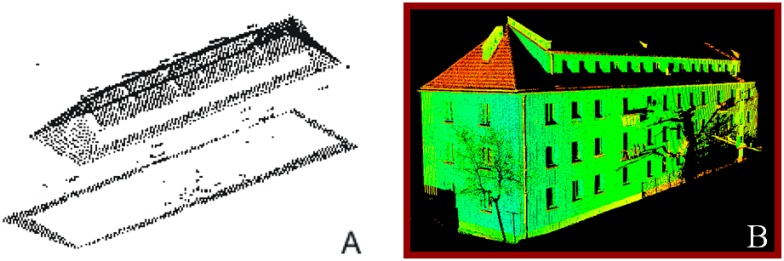
Block of flats, perspective view. (**A**) Aerial Laser Scanning data; (**B**) Terrestrial Laser Scanning data (intensity colors).

Currently, a lot of 3D models are being built on the basis of LiDAR data. Aerial Laser Scanning systems can acquire over 40 points per square meter and register multiple echos, which enables accurate terrain and surface reconstruction. However, ALS data is very often incomplete. It means there is a lack of points in some specific areas (building facade, footings, building elements located under tree cover, occlusions, *etc.*, Figure 1A). Therefore, 3D building models could be generated on the basis of terrestrial images [4], but generating models in very narrow streets using terrestrial images can be impossible. In most situations TLS can be the best source of data as it yields very dense and accurate point clouds (1–5 cm) complete with the information that is not acquired by ALS sensors (building interior, facade, *etc.* Figure 1B). Therefore, complete and accurate 3D modeling of buildings is possible by merging TLS and ALS data.

Additionally there is a question of how to merge two such different types of data: point clouds from TLS and ALS. Nowadays there is still a lack of evaluation systems that can do this task.

The objective of the proposed method is the modification and completion of ALS data set with the use of TLS data. We will show that our method increases the accuracy and density of ALS point clouds.

### 1.2. Related Work

The issue of TLS and ALS data integration is an active research area [5]. The input information can be divided into two groups: point clouds and imagery from TLS (plus mobile mapping), point clouds and imagery from ALS and 2D maps as well. Then, we can choose the specific data in specific sequences we want to combine. Many approaches include a combination of point clouds, aerial imagery and 2D maps or vector data for building extraction and reconstruction or 3D city modeling [6–8]. Compared with photogrammetric imagery, point clouds of LiDAR can give very accurate XYZ coordinates information. Nevertheless, the quality of boundary lines is quite poor. What is more, in some regions in LiDAR point clouds there might be no data. This situation is caused by self-occlusion [9]. Currently, the most popular combination is combining of point clouds with aerial images. The authors of such papers like [5,10–12] and others, find this integration has many advantages, especially in orthorectification or texturing of 3D models. Another possibility is an approach described by Kremer and Hunter in [13].

According to [14] the basic step which allows us to join photogrammetric data is the orientation to common uniform coordinate system. Rönnholm in [15] suggests a few methods of implementation of one common system for geodata coming from different measurement systems:

- usage of one hybrid tool which, after proper calibration, allows the gathering of data in one coordinate system simultaneously;
- preparation of orientation image and laser data separately but in an identity coordinate system;
- relative orientation, so called registration of different data types.

One of the methods, which allows us to make relative orientation for independently acquired data, is the transformation between systems. Transformation is based on reference points. In this case points are common for both data sets. These points could be selected among natural points in the form of roof corners, edges or other elements which make topographic objects distinctive. Target points also can be made from targets or ground signs [16].

A trend of fusion of different geodata types, which is described for instance in [17–22] has been observed for several years in the Polish and international literature. The most important conclusion from the report is a confirmation of hypothesis about the influence of both *a priori* accuracy of image and laser data and method of searching for tie points.

In all methods problems related to ALS data can be observed as a result of their “stripped” nature. The only way to adjust and to reduce the influence of those strips to accuracy of transformation is to use affine 3D transformation. There are many examples of such solutions, however, the issue of development of cities' 3D models based only on integrated point cloud from laser scanning is not so common.

In [20], a method of combining data from TLS and ALS using terrestrial and aerial scanner georeferences in a common global coordinate system was described. A terrestrial laser scanner was integrated with a low precision GPS receiver (2 m) and an electronic compass with real precision in urban area equal to 6°, which allowed researchers to get point clouds oriented to a global coordinate system with an accuracy not greater than 2 m in height and 3 m in horizontal coordinate. Moreover, during the integration of data coming from different positions, an Iterative Closest Point (ICP) algorithm was used to increase the accuracy of the general georeference. Afterwards, again with the application of the ICP algorithm, oriented TLS data was joined to DTM and 3D models of buildings were generated from ALS point cloud and cadastral data. Then, the general orientation of TLS scans could be used as a basis for an iterative solution to adjust the two sets of data. Results and analysis of the precision of such solution are described in detail in the mentioned publication.

In the case of integration through data orientation based on the direct transformation of sets of points to common coordinate system (usually global system defined by ALS). Coordinates are recalculated using a specific function with parameters based on reference points. These points are selected among so-called tie points or pseudo-homological points. Then the basic problem is to find such points with high accuracy. Depending on the entry data available, we could distinguish general orientation (a technique used to estimate parameters of transformation between two data sets without knowledge of initial reference points) and detailed orientation (when initial parameters of transformation are already known). General orientation is considered as one of the most popular methods of joining 2D or 3D data sets. The main assumption of such an approach is to define the identity and characteristic elements of the subsets joined. A common area of cover is then required. In such an area some characteristic elements can be distinguished such as points, straight lines [23], curves [24], planes, figures, *etc.* Another method is an algorithm, described by Chua [25], which defines signature of point. A selected point is described indirectly as a vector calculated by other points located in its neighborhood (defined by particular distance). Then, all selected points are compared with the second set in order to find identity points. In most cases, the accuracy of general registration is not enough to be used in the photogrammetric field. That is why detailed methods are used. However, in order to apply them, initial knowledge about general or precise information about reference points or parameters of transformation has to be available. Common iterative methods are used, which are based on minimizing defined values, like transformation error or defined physical parameters, e.g. the distance between points.

## 2. Description of the Issue

An analysis of existing knowledge and possibilities of geodata usage found that integrating data from terrestrial and aerial laser scanning has to be based on the transformation of terrestrial laser scanning data from the local coordinates system to the global coordinates system of aerial laser scanning data. That is why the main task was to find proper transformation parameters based on reference points. Therefore, proper definition and selection of tie points in both data sets was the basic issue.

On the basis of current research and analysis it was concluded that the main problem of integration is identifying and selecting proper (*i.e.*, accurate and unambiguous) characteristic points, which will be considered as reference points used to perform the transformation between TLS and ALS coordinate systems. The main problem here is the nature of LiDAR data that means “point cloud” and has a great influence on point identification and interpretation. In the figure below, an example of different geodata character and its consequences is presented.

**Figure 2.**
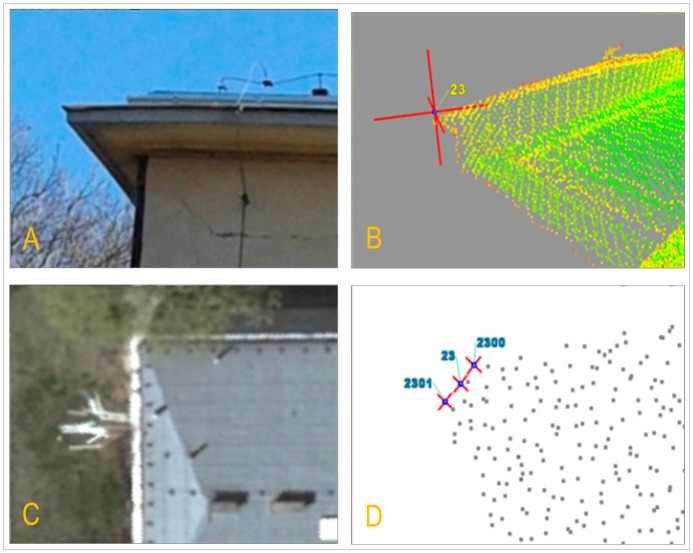
The view of building edge. (**A**) terrestrial image; (**B**) TLS point cloud perspective view; (**C**) aerial image; (**D**) ALS point cloud.

Figure 2D represents three possible location which are subjective interpretations of a building's corner location in ALS data. The same object in an aerial image is shown in Figure 2C.

It is almost impossible to define either an exact point representing the building's corner or the identity of this point in both TLS and ALS data sets. This is a basic step in the process of analysis and it includes the verification of different methods for points identification. We distinguish direct and indirect methods.

Direct methods are based on a manual point selection, for example by visual interpretation of the building's edges and corners in a point cloud. Indirect methods mean automatic or semi-automatic definition of pseudo-homological point coordinates, using data processed at different levels (filtration, classification, 3D model). The authors are proposing a new method with a method using wavelet analysis to increase accuracy of ALS data and, along with it, increase the precision of finding characteristic points. The results were compared with the direct and indirect method using a simple regression model.

### 2.1. Methods of Characteristic Points Extraction

In this article a new method of defining coordinates of characteristic points using wavelet analysis is described. Initially, selected ALS and TLS points representing roof edges are the basic component of the algorithm.

#### TLS Data—Identification of Edge Points

**Figure 3.**
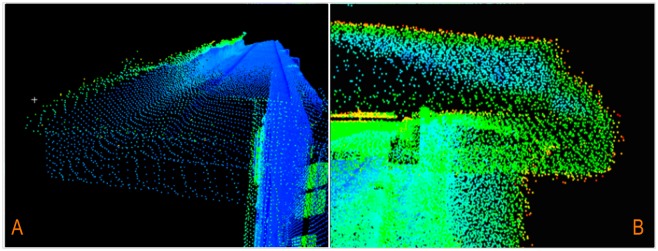
The view of the building's corner. (**A**) Corner of tested building (phase laser scanner data presented in intensity colors); (**B**) The same corner of tested building (pulsed laser scanner data presented in intensity colors).

Points belonging to the edges of the roof can be made from different material than the rest of the building elements. Therefore, their intensity will be different. Figure 3A,B shows differences in intensity for a selected corner of the building both for phase and pulsed laser scanner data.

In the case of the example presented in Figures 3, the value of reflection intensity for the edge of the roof is in interval 0.27–0.69 for data from the phase scanner and 0.12–0.28 for the pulse scanner. Filtration based on intensity allows the selection of points, which probably (80%–90%, depending on the type of data and object) satisfies the condition of belonging to peripheral elements: edges and adjacent elements like vegetation, installation, *etc.* Oriented and integrated data were filtered and classified.

### 2.2. ALS Data—Identification of Edge Points by Indirect Method Using Wavelet Analysis

This kind of integration is based on the transformation between high resolution TLS data and low accuracy ALS data. The extraction of a signal characteristic can be done mathematically and analytically via approximation, averaging, examination of signal's parts or transformation—herein Fourier transformation.

Wavelet analysis represents a signal using hierarchical resolution to receive information about the signal that can be presented in different levels of details. It extracts a general approximation but also enables the selection of some details, disturbances or noise. Theoretically, the variability of relative locations of adjacent points representing the roof edge should be exactly the same in both cases. The main aim of this research was to define whether it is possible to “improve” aerial laser scanning data based on data from terrestrial laser scanning.

Wavelets are sets of functions where each of them is extracted from basic function using shifting and scaling. Generally speaking, wavelet analysis can be described as [26,27]:

(1) $$\gamma \left(s,\tau \right)=\int^{}f\left(t\right)\psi_{s,\tau }^{*}\left(t\right)dt$$

where * is the complex conjugate. Function *f(t)* is decomposed to separate functions (wavelets):

$$\psi_{s,\tau }\left(t\right)$$

Variable *s* is a scale and *τ* is shift. Then, reverse transformation could be described as:

(2) $$f\left(t\right)=\iint \gamma \left(s,\tau \right)\psi_{s,\tau }\left(t\right)d\tau ds(2)$$

Using wavelet analysis, decomposition of signal and its lossless reconstruction is possible [26].

#### 2.2.1. Discrete Wavelet Transform—DWT

In case of discrete wavelet analysis, the signal is scaled and shifted discretely, which we could write:

(3) $$\psi_{j,k}\left(t\right)=\frac{1}{\sqrt{s_{0}^{j}}}\psi \left(\frac{t-k\tau_{0}s_{0}^{j}}{s_{0}^{j}}\right)$$

where *j* and *k* are integers and *s*_0_ > 1 is step of extension, which has impact to shift *τ*_0_ (it is considered that *τ*_0_ = 1). Usually factor *s*_0_ = 2, so sampling of frequency could correspond to dyadic sampling (power of 2).

**Figure 4.**
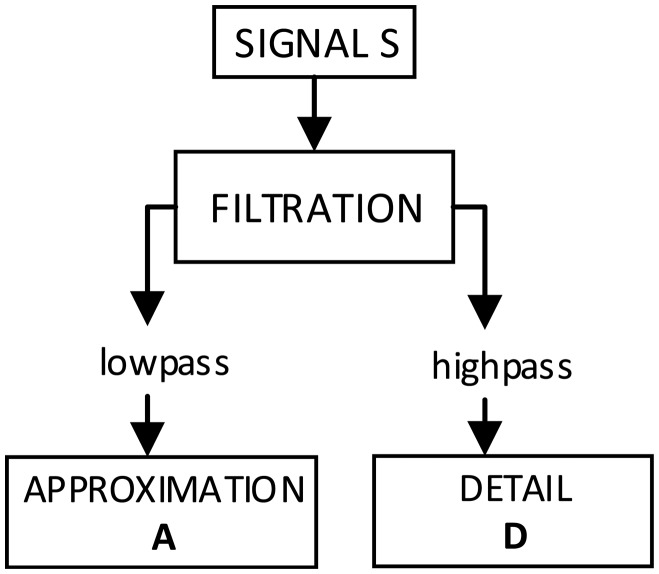
A scheme of wavelet expansion using filtration.

In DWT, the wavelet could be presented as a high-pass filter. This fact is used in wavelet expansion of signal in multi-resolution structures. Such expansions could be found using a discrete algorithm by means of multistage set of filters. An algorithm based on this dependency is called Mallat's algorithm[27–29]. A scheme of wavelet expansion in simple form is shown in Figure 4.

The wavelet is shown as a high-pass filter, thereby generating details of signal D, whereas the low-pass filter (general information about signal, approximation A) is defined by scaling function, determined by coupled reflex filters. Part A could be expanded again (Figure 4).

#### 2.2.2. Signal Reconstruction

**Figure 5.**
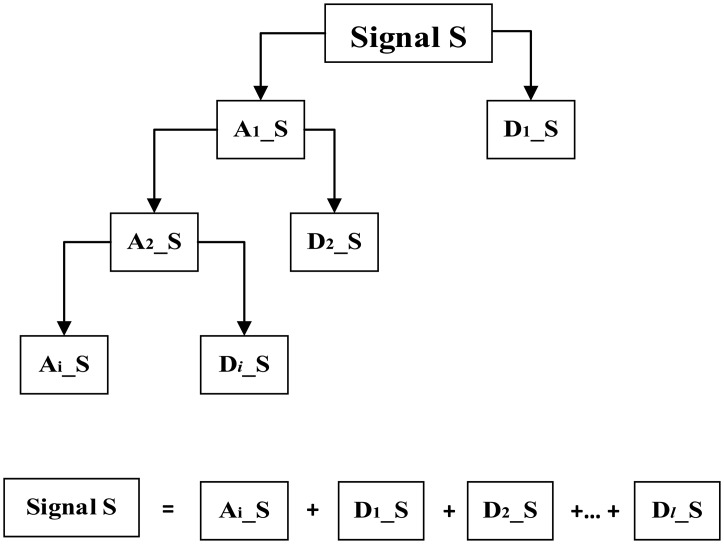
Scheme of signal reconstruction from approximation (A) and details (D).

The reconstruction of a signal is a process which is reverse to its expansion by decomposition and it is named Inverse Discrete Wavelet Transform (IDWT). The reconstruction scheme is shown in Figure 5. The aim of the reconstruction is to create an original signal by a recursive addition and subtraction of the coefficients of details' coefficients and of average values corresponding to higher resolution.

#### 2.2.3. Processing of Edges

The edge reconstructed only from ALS data is encumbered with relatively high error having a value close to the average distance between points. Even if the edge contains no classified points, or they are improperly classified, it could be approximated with a straight line or be made more accurate based on different data, like image data or other point clouds.

In this section an entirely new method will be proposed, which relies on laser scanning data processing by means of wavelet analysis. The aim of the processing is to increase ALS data quality, which results in a better projection of edge and location of particular points of edge in ALS data by means of TLS data. Because of the nature of data character, Discrete Wavelet Transform was used. The method proposed is independent of type and definition of both signals' coordinate systems.

**Figure 6.**
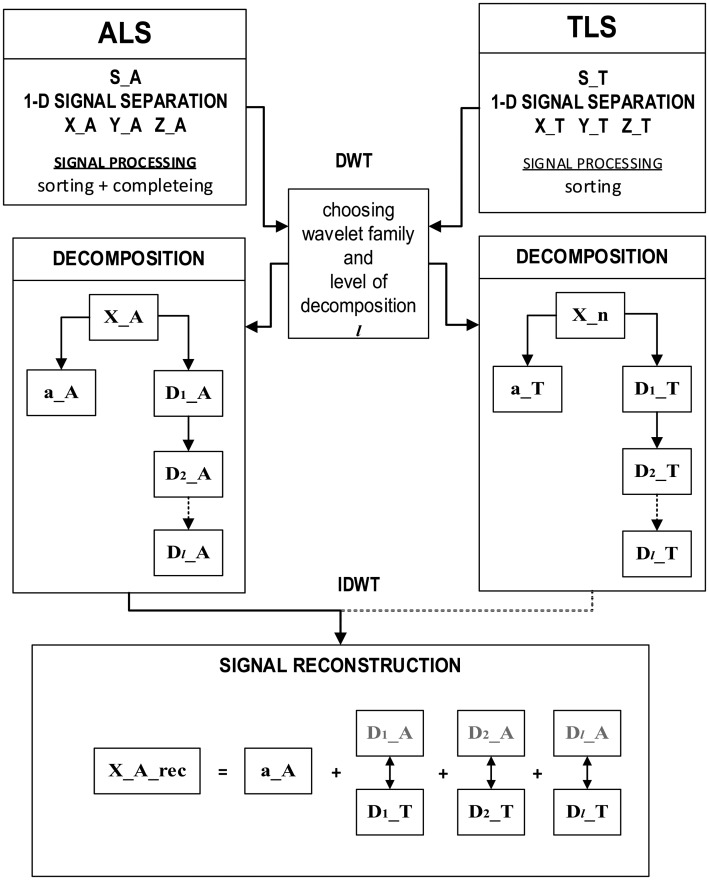
Detailed scheme of the algorithm.

Generally speaking, this method assumes a detection of high-frequency changes of signal, which correspond to details of scanning data, separately: TLS and ALS. After the extraction of the characteristic elements (details) of the signal proceedings in the frequency domain, a reconstruction of the signal is made. Features (details represented by high frequencies) of TLS data are assigned to features (details) of corresponding ALS data. Figure 6 presents a general schema of the idea:

In the presented figure the meaning of the symbols is: S_A and S_T—signal of ALS and TLS point cloud data, D_A and D_T—details of ALS and TLS data; a_A and a_T—approximation of ALS and TLS data.

All signals representing individual coordinates for edges' data were analyzed using wavelet analysis. Each signal (point series) was decomposed to *l* levels in different intervals of details and to a component called approximation (a_A—approximation of aerial data). Then, by sum of details and approximation the signal was reconstructed.

### 2.3. Algorithm Description

The algorithm specifies the main stages of data processing described below:

#### 2.3.1. Selecting the Edges and Splitting into One-Dimensional Signals

The first step is to select points belonging to the edges independently for TLS and ALS data sets by using any method and to save them as two separate three-dimensional signals. Then, components of X, Y and Z have to be separated into one-dimensional signals and be saved as individual vectors **X**, **Y** and **Z**.

#### 2.3.2. Aligning the Length of the Signal (Completing ALS Data)

In an analysis of a one-dimensional signal it has to fold up from points arranged in a particular order. Data has to be then sorted, if it had not been done earlier.

Sorting has to be done at once for all coordinates by one of them. It is especially important in the case of defining connection and identity of the following triples **X**, **Y** and **Z** to unambiguous identification and defining couple (or triples) of coordinates as one point.

The resolution (higher distances between points) of ALS point cloud is about ten times lower than TLS. Moreover, in 95% of the cases the ALS edge is shorter than the TLS one. This means, missing data have to be filled in for the resolution and length to be similar in both point clouds.

**Figure 7.**
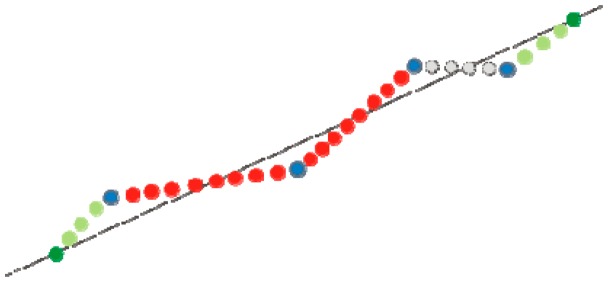
Algorithm of sorting and completing edge points, Method 1.

There are several methods of adding points “between existing ALS points”. Below, two methods developed by the authors are presented, which allow the transformation of ALS point clouds in a way which makes them usable to analyze along with point clouds acquired by TLS. Authors propose two methods of completing data based on elementary edge points and location of original ALS points via linear approximation and uniform location of points between original points (Figure 7).

(a) Method 1:
  The algorithm is as follows:
  (1) Sorting points by one of the coordinates.
  (2) Calculation of difference between number of points from TLS and ALS, that gives number of points to add *L_p_*.
  (3) Calculation of signal range. For data sorted minimal value is subtracted from maximal value.
  (4) Calculation of difference between length of ALS and TLS segments *Δ_LN_*.
  (5) Processing of data, addition:
    a. Determination of parameters of a straight line *p_a_* 2D approximation of real points (X,Y).
    b. Extension of edge: adding two new peripheral points: each of them in distance $\frac{\Delta_{LN}}{2}$ from original peripheral points. These points are added based on equation of line *p_a_* and since that time are considered as real points.
    c. Interpolation of points.
    a. Points are added iteratively between each pair of consecutive (neighbouring) real points according to the following algorithm:
      i. Calculation of distance between each pair of consecutive points *d_pp_*.
      ii. Calculation of the number of points *k* added between two real points, by calculating the proportion between distance *d_pp_* (found in previous points) and secondary length of the whole edge (segment) *d_L_*:
        (4) $$k=\frac{d_{pp}}{d_{L}}\cdot L_{p}$$
      iii. Calculation of distance between added points *d_ap_*:
        (5) $$d_{ap}=\frac{d_{pp}}{k}$$
      iv. Calculation of the coordinates of points added:
        - Determination of a straight line equation passing by two consecutive (neighbouring) real points.
        - Determination the X and Y coordinates of points, added in number of *k*—based on line *p* equation.
  An example showing the implementation of Method 1 is presented in Figure7.
  Blue dots represent the original ALS data, red dots are points added between the original points, green dots are peripheral points and points between them.
(b) Method 2:
  The algorithm is as in the first method from Part 1 to 5iii. The difference appears when calculating the coordinates of added points (5iv).
  iv. Calculation of the coordinates of points added:
    Determination of a straight line equation passing by real points
    Determination the X and Y coordinates of points—added in number of *k*—based on line p equation.

**Figure 8.**
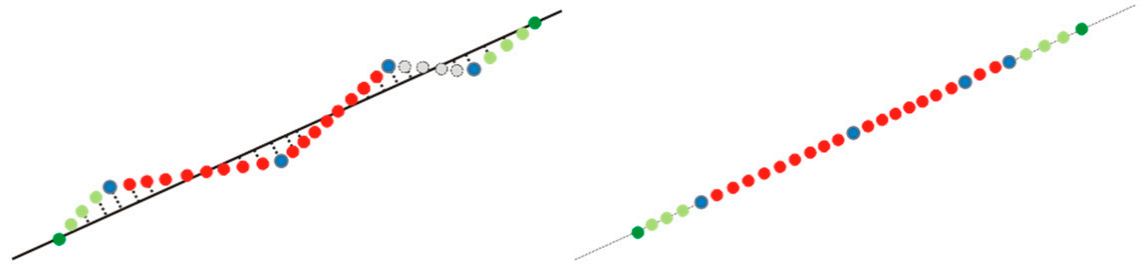
Algorithm of sorting and completing edge points, Method 2

An example showing implementation of Method 2 is presented in Figure 8.

Blue dots circles represent the original ALS data, red dots: points added between original points, green dots: peripheral points and points between them. The described algorithm and procedures were implemented in the C# environment.

#### 2.3.3. Single-Level and Multi-Level Decomposition

As mentioned before, a signal could be decomposed into the low-frequency part (approximation) and the high-frequency part (details). For many of the signals the most important element is the low-frequency part, which determines the general characteristic of signal tendency. The high-frequency part corresponds to the detailed nature of the signal and enables separating noise from the signal.

In this method, the important part are details, which describe the detailed character of data (D_T—details of terrestrial data, D_A—details of aerial data). All signals representing individual coordinates for the edges' data were analysed using wavelet analysis. Each signal (point series) was decomposed to *l* levels in different intervals of *details* and to a component called *approximation* (a_A—details of aerial data). Then, as a sum of details and approximation the signal was reconstructed.

Both single-level and multi-level decomposition were analysed. The aim of the analyses is to verify which type of signal decomposition and wavelet family is adequate for the signals examined, and how they have been split into frequency intervals.

#### 2.3.4. Determination Wavelet Family and Level of Decomposition

Decomposition, depending on data, can be single- or multi-level. It is assumed, that the level of decomposition *l* is dependent on the length of the signal (series). There are only a few methods which allow defining the level of decomposition for signal, and often it is theoretical or empirical estimation. The main way to define it is by means of entropy [30]. Length of the signal is usually key information for the level of decomposition to be chosen.

Each level of decomposition causes decomposition of the current approximation into another signal of approximation and details at next level (Figure 6). Choosing the wavelet family and level of decomposition is an empirical issue. When the type of wavelet and its similarity to original signal are known, then the level of decomposition could be defined. When the type of wavelet has to be chosen, shape and features of the signal will be taken into consideration. The main criterion is similarity (correlation) between the original signal and the shape of the selected wavelet.

The most important thing in wavelet analysis are details, both those with high and low amplitudes at different decomposition levels. It is important, because appropriate subtractions and change of particular details will be implemented at separate signals, however used together when reconstructing signals [31].

#### 2.3.5. Signal Reconstruction

A signal can be reconstructed to its full form by IDWT. Generally it is based on the composition of a signal by the addition of adequate wavelet coefficients, approximation and trend. The basic rule of mixed reconstruction is the invert decomposition of ALS signal taking into account decomposition coefficients of TLS and ALS signals (Figure 6).

## 3. Empirical Study

### 3.1. Data Sets

**Figure 9.**
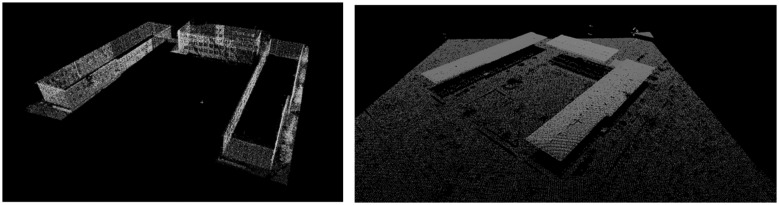
TLS data set (on the left); ALS data set (on the right).

The data used in our research were acquired using TLS and ALS systems (Figure 9). The TLS data sets were acquired using a Leica ScanStation2 in the Military University of Technology area, in the city of Warsaw, Poland, in 2010. This is a pulsed HDS scanner with a range of 300 m and high measurement accuracy (position: 6 mm, distance: 4 mm and high scan resolution below 1 mm). The system is equipped with a terrestrial digital camera (6.4 Mpx). The main issues related to terrestrial laser scanning data acquisition are related to range and incidence angle, that determines indirectly how many points in the upper parts of the building (for example roofs) will be collected. The RMSE of the measured points coordinates was within the range of 3 to 8 cm. To compare we used also a FARO Focus 3D phase laser scanner.

The ALS data sets were acquired in 2009. Scanning was conducted using the ALS Lite Mapper 6800i with a 4–6 points per square meter resolution. ALS measurements data usually consist of not only points information from the top surfaces of the structures (buildings' roofs, the canopy of trees) but also from some branches and lower parts of the trees. This feature will be further named as data incompleteness. Also the merging of objects is very common. The accuracy of measurements was determined using check points (RMSE < 0.30 m in altimetry and 0.10–0.50 m in horizontal error).

The test field consists of buildings with different roof types (flat, steep, arched, *etc.*) and different structures.

### 3.2. DWT Signal Processing

**Figure 10.**
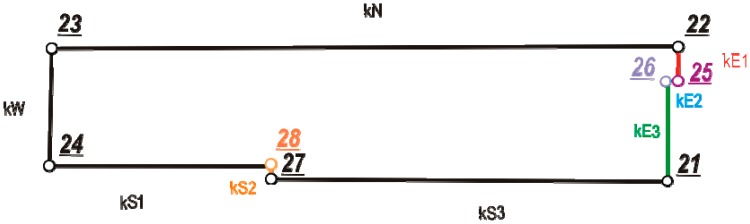
Sketch of test building with labeled vertices and edges.

Figure 10 presents data divided into vertex and edges with descriptions (*k*—means edge for one of test building).

**Figure 11.**
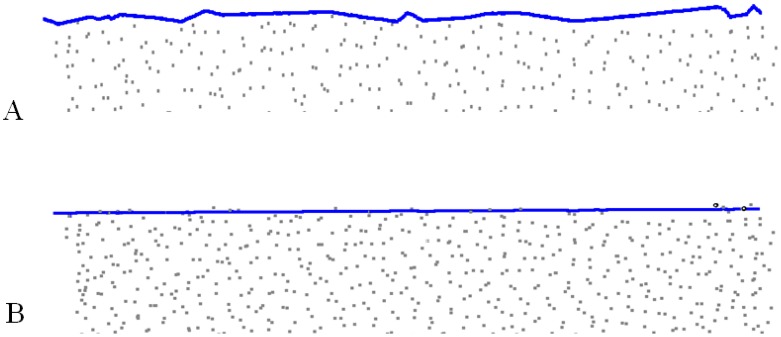
Results of completing edge points: (**A**) Method 1; (**B**) Method 2.

Below there is an example of edge kW processing. In the first step, the alignment of the signal was conducted. The results of processing using two proposed method are showed in the Figure 11A,B.

In Figure 11A,B, points in blue represent completed edge data and points in gray present original ALS points. In both cases, the number of edge points is the same. The difference is invisible in the linear and non-linear data character. Below we give an example where the X coordinate of a signal is decomposed for selected edge kW of aerial (kW_A) and terrestrial (kW_T) laser scanning aligned with Method 1. Signals kW_A and kW_T were analyzed in different variants (wavelet family, level of decomposition).

#### 3.2.1. Selected Wavelet: “sym3”, Level of Decomposition: *l* = 8: “8 sym3”

**Figure 12.**
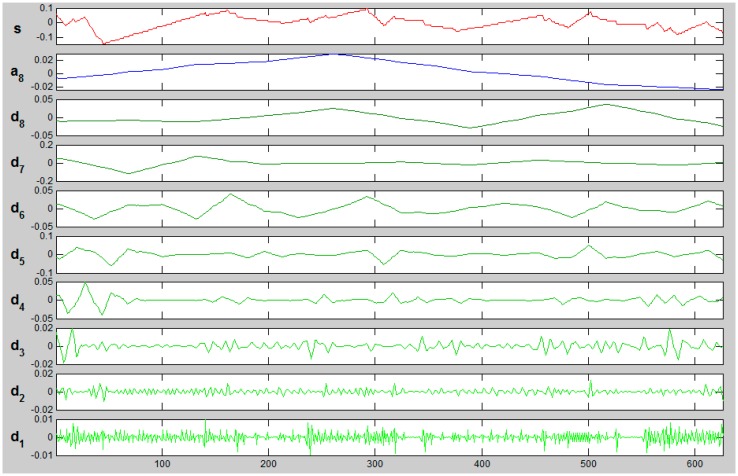
Signal decomposition with wavelet “sym3”, eight levels.

**Figure 13.**
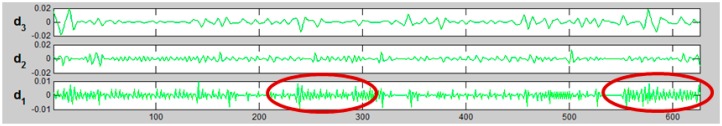
Details of d1, d2 and d3 of ALS signal decomposition, wavelet “sym3”, eight levels.

First family of wavelets taken into consideration is Symlet: wavelets “sym2” and “sym3”. Figures 12 and 13 present exemplary decomposition of signals representing X coordinate of kW edge from ALS.

**Figure 14.**
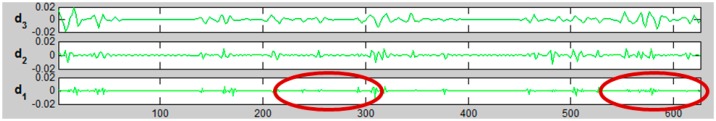
Details of d1, d2 and d3 of TLS signal decomposition, wavelet “sym3”, eight levels.

Figure 12 presents the decomposition of ALS signal (kW_A) using “sym3” wavelet for eight levels. S stands for signal, a—an approximation, d_i_—details. When extracting the characteristic of edge “noise”, details d1, d2 and d3 contain relevant information. Figures 13 and 14 present a comparison of d1–d3 details for the same edge from the ALS and TLS data, respectively.

The biggest differences could be observed for d1 and d2 in details, both in TLS and ALS data (marked with red ellipses). In case of d3 details, differences were rare and insignificant. The next considered set of wavelets was Daubechies wavelet “db2” level 8.

#### 3.2.2. Selected Wavelet: “db2”, Level of Decomposition: *l* = 8: “8 db2”

**Figure 15.**
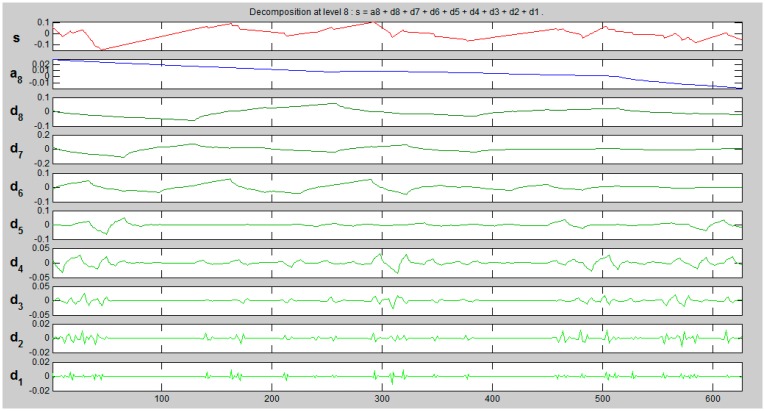
ALS signal decompositions with wavelet “db2”, eight levels.

Figure 15 presents the decomposition of ALS signal for eight levels with the “db2” wavelet.

Both decompositions were quite similar. As it could be seen (Figures 12 and 15), details from the decomposition with wavelets “sym2” and “db2” for the same signal had the same character and almost the same changeability. Differences could be observed already in d6 details when comparing “db” with “sym3” wavelet. The reason of that is mainly because Symlet wavelets are a modification of Daubechies wavelets. Other wavelet families like Coiflet and Mexican hat wavelets were also analyzed. Taking into consideration the characteristic of point clouds, finally “symlet3” had been chosen. In general we can say that every signal should be analyzed individually depending on its character and length. The other edges were analyzed in the same way. Decomposition for each of them was selected separately and further steps were defined.

### 3.3. Reconstruction of Signal and IDWT

As an example of mixed signal reconstruction, we chose another, shorter signal kE3 and found the best decomposition with wavelet “sym3” at level *l* = 6. For instance, to reconstruct a signal, approximation of original ALS signal from level 6 was used along with details of TLS signal. It could be then understood as “making noise” for the ALS signal based on the TLS signal. The observation of the decomposed signal and its wavelet coefficients allowed us to conclude the following possibilities of reconstruction using an A—approximation of ALS, dT—details of TLS, dA—details of ALS:

(a) aA6 + dT1 + dT 2 + dT 3 + dT 4 + dT 5 + dT 6
(b) aA 6 + dT 1 + dT 2 + dA3 + dA 4 + dA 5 + dA 6
(c) aA 6 + dT 1 + dT 2

**Figure 16.**
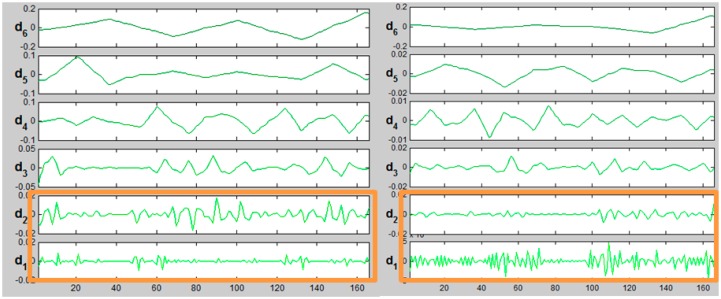
Details of d1–d6 respectively for XA_kE3 (ALS on the left) and XT_kE3 (TLS on the right); “sym3”, level 6.

Figure 16 presents d1–d6 details for signals of the X coordinate from aerial (XA_kE3) and terrestrial (XT_kE3) point clouds representing the kE3 roof edge. Decomposition was made with sym3 wavelet at level 6.

The difference between both signals as it could be noticeable at lowest frequencies was largest for details d1 and d2 (Figure 16). These differences indicate the different density of “points of roof edge”. Signal reconstruction (XAT_kE3_6sym3_a6d12) was done using approximation a6_XA (coarse ALS signal representation) at level 6, details from ALS: dA6, dA5, dA4, dA3 and details from TLS dT2 and dT1:

**XAT_kE3_6sym3_a6d12** = a6_XA + d6_XA + d5_XA + d4_XA + D3_XA + **d2_XT + d1_XT**

**Figure 17.**
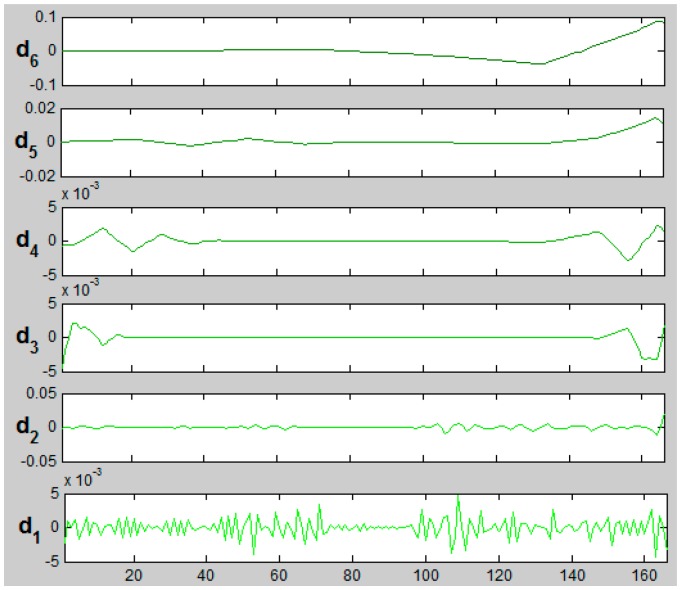
Details of d1–d6 of reconstructed signal X_AT_kE3_6sym3_a6d12.

The signal of X coordinate of kE3 edge was decomposed with the same wavelet “sym3” at level 6. The results are depicted in Figure 17.

**Figure 18.**
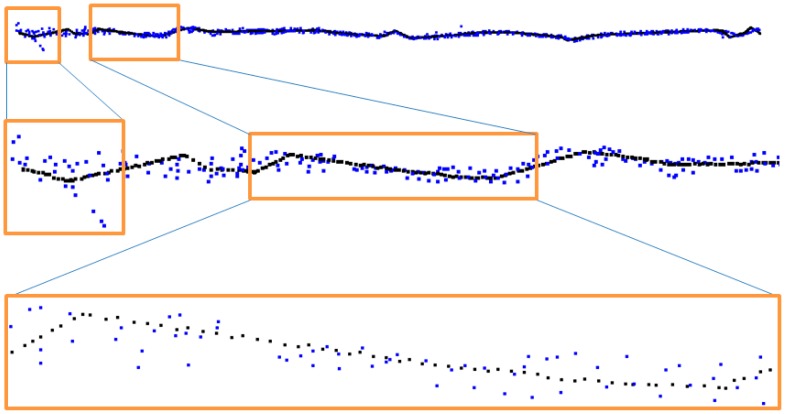
Points representing kE3 roof edge after mixed reconstruction.

**Figure 19.**
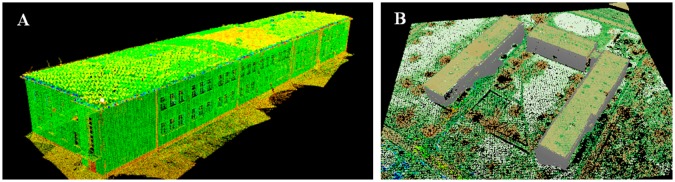
Integrated ALS and TLS point clouds (**A**) One separate test building; (**B**) Complex of buildings.

Differences could be seen not only for d1 and d2 details but also for higher details. Analysis was made for all coordinates X, Y, Z separately. Finally, the signal of a complete kE3 edge was reconstructed taking into account details d1 and d2 from the terrestrial laser scanning data and presented in Figure 18. The points marked with black colour represent data before decomposition, blue colour represent data after decomposition. More detailed views of edge points are presented in consecutive layers in Figure 19.

Similarly, decomposition and reconstruction were performed for all coordinates for all other edges of test building using both first and second methods of ALS data completion. After edge reconstruction, the vertices of the roof were determined as an edges intersection. These vertices were used as a reference, homologous points in further transformations.

## 4. Integration by 3D Transformations

The aim of the above mentioned data processing was to increase the accuracy of characteristic point identification. We can say that the better the reference points accuracy, the more accurate a transformation is. What is more, proper integration needs proper transformation between coordinate systems. There are conforming (linear) transformations and polynomial transformations of higher degrees, which should be mentioned here. Analyses were performed using 3D conforming, isometric and affine transformations. Analysis was based on reference points found by direct method (raw ALS data) and wavelet analysis.

The analysis of selected tie points after transformation was based on the comparison of transformation errors and deviation for reference points. In the case of lower number of reference points, the general error of transformation was also lower, however deviations for reference points and check points were risen to about 30%. In Table 1, results showing accuracy of transformation have been presented. Analysing Table 1, it could be noticed that the general mean error of transformation had the highest value for methods based on raw data, independently of the type of transformation, which is correct with the predictions made.

The accuracy of transformation performed using reference points taken from “wavelet-reconstructed” data rises almost two-fold when compared to the direct method. In all cases, the average error of transformation was lower for conformal transformation (the one using change of scale). The results of fusion of cloud points from TLS and ALS based on conformal transformation are presented in Figure 19A,B.

Figure 19A presents a perspective view of integrated point clouds in intensity values for one single test building. Figure 19B shows a complex of three buildings as a result of point cloud fusion. The gray color represents TLS data, and the green part represents ALS data processed by using wavelet analysis.

## 5. Discussion

The presented experiments and their results from Sections 3 and 4 show the usability of proposed method. The method was tested on a real data set. The methods of edge completion (Section 2.3.2) are independent of edge length or shape. Generally, better results are achieved in the second processing (Method 2). This is caused by “linearization” of the edge. However, in the authors' opinion, methodically, the second method is a better solution. The noise and real data character are real and not tuned.

The main drawback of the presented wavelet method is the quite time-consuming determination of level of decomposition and wavelet family. These two factors strongly depend on data and need to be determined semi-automatically and often empirically. The wavelet family should correspond to the data characteristics, especially for point clouds. On the basis our research and literature studies, the most suitable are the Symlet and Daubechies families. When using wavelet analysis, the fact that the signal (points series) should have clearly diversified energy is very important. It means that signals should have quite big amplitudes, so that it will be possible to use them as a detailed characteristic of the signal. However, the amplitude in the last levels of decomposition should not be constant or too small. In such a situation there will be no possibility to extract relevant characteristics of the signal, also decomposition of higher number of levels would have no sense. What is more, these levels of decomposition with white noise should not be considered. When defining the decomposition level and amount of information in particular levels, quantitative division of coefficients is important. It means that depending on the number of levels the number of coefficients in particular (decomposition) is decomposed according to geometric sequence. For instance, when having a six level decomposition, 50% of coefficients are accumulated in the first level, 25% of them are in the second, the third contains 12.5% of them and in the sixth there are 1.5% of them.

### 5.1. Quality of the Method

Additional analysis was performed to examine the quality of the proposed method of pseudo-homologous point determination. Table 2 presents the list of edges and segments (column: Edge), lengths (column: Reference Measurement T) calculated on the basis of tachometric measurements and absolute values for edge length differences (column: Difference). These differences were calculated between reference measurement and raw ALS edge data (T-ALS_D), ALS edge data after wavelet reconstruction using first and second method of completing the signal (AP_VA I and II) and edge from TLS data (TLS).

The absolute mean difference value is presented in the last row of Table 2. For edge lengths calculated directly from raw ALS point cloud, the mean value is about 20% bigger than the length from data processed using wavelet analysis. In comparison to particular length values the results are similar. Only two shortest edges of the building cannot be processed using described method. In ALS data the biggest difference does not exceed value of 0.645 m, after wavelet analysis, 0.107 m for the same edge, and 0.427 m generally. Compared to the rest presented methods, where the time of data processing was much shorter, the errors of final transformation were much smaller.

The method was implemented with the MATLAB Wavelet Toolbox. The input data came from point processing according to the method described in Sections 2.1.1–2.3.2. Depending on the chosen method, edge processing is manual or automatic. Our method is semi-automatic, because it needs initially prepared input data. If edges are primarily prepared, the only manual step is determination of wavelet analysis parameters.

The computational cost of the proposed method depends on the data preparation level and quality. For example for unfiltered data there is a need to separate point edges from noise. This can be done manually or automatically. The most time-consuming step is the edge identification and the alignment of the signal. With the implemented algorithm, for one edge it takes about 10–20 min to extract, complete and load data to a MATLAB application. Further steps' time depends on the knowledge and an experience of operator and complexity of data and could take a few minutes. All processing and implementations were made using 2.50 GHz Pentium processor with 4.00 GB RAM and 64-bit WOS.

## 6. Conclusions

Many approaches of multi-sensor data integration include the combination of point clouds, imagery and 2D maps or vector data for building extraction and reconstruction or 3D city modeling. However, many of them are not accurate enough and do not apply the highest TLS measurement accuracy. The main issue of discussed data integration methods was an adequate reference point selection from the multi-resolution laser scanning data. This paper proposes a new method of using wavelet analysis in the reconstruction and identification of tie points, which did not existed in an original airborne LiDAR data set (because of its resolution). The results of experiments have shown that by using the proposed method the ALS and TLS point clouds integration can be improved in relation to classical methods. The existing data integration approaches have also been analyzed taking into consideration the type of transformation, tie points identification methods and their location in space. Further research will be developed by using specific types of wavelets in LiDAR data integration and taking into consideration particular structures and objects types. In the future work, comparisons of our method with other available methods with a larger number of objects will be undertaken. Also, the level of automation of the presented method will be improved.

## Figures and Tables

**Table 1.** Accuracy of transformation.

	**Transformation Mean Error (m)**	

	**Isometric**	**Conformal**
direct method	0.471	0.431
wavelet analysis	0.218	0.203

**Table 2.** Absolute differences in segment lengths respectively reference measurements.

**Edge**	**Reference Measurement T (m)**	**Difference (m)**			

		**T-ALS_D**	**T-AP_VA I**	**T-AP_VA II**	**T-TLS**
23–24 kW	15.588	0.210	0.250	0.250	0.000
23–22 kN	81.069	0.344	0.128	0.053	0.010
22–25 kE3	4.617	0.645	0.107	0.048	0.036
21–27 kS3	51.428	0.498	0.427	0.098	0.088
27–28 kS2	1.634	0.002	0.200	0.156	0.021
28–24 kS1	28.620	0.144	0.217	0.301	0.047
22–21	17.491	0.625	0.347	0.291	0.097
25–21	12.909	0.006	0.271	0.275	-
27–24	28.670	0.145	0.251	0.251	-
Mean (m)	-	0.291	0.244	0.191	0.043
